# Supplementary material for: Modelling response strategies for controlling gonorrhoea outbreaks in men who have sex with men in Australia
Source: PLoS Comput Biol. 2021 Nov 4;17(11):e1009385. doi: 10.1371/journal.pcbi.1009385 (PMC8594806; doi:10.1371/journal.pcbi.1009385)
Supplement: Attachment — Submitted filename: PLOS Computational Biology_March_21.docx [file pcbi.1009385.s002.docx]

**PLOS Computational Biology – PCOMPBIOL-D-21-00242**

**Modelling outbreak response strategies for preventing spread of emergent Neisseria**

**gonorrhoeae strains in men who have sex with men in Australia**

**Comments**

This is a modelling study evaluating the impact of several public health strategies (such as different screening rates combined with subsequent treatment, or different contact tracing levels) on the dissemination of emergent or imported strains of Neisseria gonorrhoeae in Australian MSM. The authors find that the current approach (STI screening 1x/year, unless more than 20 partners/year) will not succeed in eliminating emergent or imported NG strains, unless response strategies are combined (e.g. increased STI screening + contact tracing of casual partners).

The manuscript is well-written, and the methodology seems sound. I have however a few concerns around the applicability, the reporting, and the reproducibility of the study findings.

1. If the aim of this paper is to model different strategies so as to better inform public health authorities and help shaping new guidance, then the model should reflect the current situation in Australia. In the light of the recent, high uptake of PrEP programs (EPIC-NSW or PrEP-X) by a relevant part of the MSM population, I am a bit concerned whether the present model structure really allows up-to-date inferences to be made. The use of PrEP has been suggested to affect several of the model parameters, such as condom use, casual partner acquisition rate or the distribution of the model population into high (27%) and low (73%) risk. It would be thus useful to adapt the model so that it reflects the changes induced by the wide implementation of PrEP programs. As a minimum, I would strongly suggest to update the parameter “casual partner acquisition rate”, which refers to data from the Health in Men study (published more than 10 years ago).

2. Throughout the manuscript (including key words and abstract), there is a lot of emphasis on drug resistant NG strains, so that the reader truly expects a model estimating the effect of different response strategies on a resistant type of NG. Surprisingly, this aim is not further pursued in the analytical part and the reasons given in the discussion seem to reflect some post-hoc decision. It would be very useful to provide a study protocol describing how the analysis was originally planned, and detailing the changes that were made to the initial plan.

3. It is not clear if someone can switch from casual partnership to regular partnership, and vice versa once they separate from their last partnership. It is also not clear if the NG strain is imported only once or whether it is a continuous trend? This should be specified. Assuming there is no switching of partnership type, it is a bit surprising to see that partner testing in those with stable partnerships only would also result in a complete extinction of NG strains in those with casual partnerships. One would rather expect an endemic situation in the cluster of those having casual partnerships only. How do you justify this?

4. ‘$\text{Pharynx :urethra: rectum=49:17:34}$’ has been simplified to ‘$\text{pharynx: urethra: rectum=}\text{47:23:3}$’. I assume the last number (i.e. 3) is a typo. If not, the simplification would be totally different from the ratio derived from ACCESS database. Please explain.

5. There is no information provided on the program/software used. It is necessary to provide this information for reproducibility. In addition, please provide a link to the source code of your model on a GIT repository.

6. Figure 1 seems a bit jumbled and hard to understand as there is no transition between the states such as ‘disease transmission’ and ‘recovery’. Also, it is worth mentioning SEIR in bracket in ‘diseases transmission’

7. In table 9, it is unclear why you are presenting the column ‘Calibrated solution (worst)’? Rather provide the best solution and 95% quartile or CI around the best calibrated solution.

8. Figure A5 of supplementary is exactly the same as Figure 3 panel B and C in the manuscript. I suggest you remove one of them.
