# Supplementary material for: Modelling response strategies for controlling gonorrhoea outbreaks in men who have sex with men in Australia
Source: PLoS Comput Biol. 2021 Nov 4;17(11):e1009385. doi: 10.1371/journal.pcbi.1009385 (PMC8594806; doi:10.1371/journal.pcbi.1009385)
Supplement: Attachment — Submitted filename: Responses to reviewers v3.docx [file pcbi.1009385.s003.docx]

Response to Reviewers

Dear Prof. Roger Dimitri Kouyos and Prof. Virginia Pitzer,

Thank you for giving us the opportunity to submit a revised version of the manuscript “Modelling outbreak response strategies for preventing spread of emergent Neisseria gonorrhoeae strains in men who have sex with men in Australia (PCOMPBIOL-D-21-00242)” for publication in PLOS Computational Biology. We appreciate the time and effort that you and the reviewers dedicated to providing feedback on our manuscript and are grateful for the insightful comments on our paper. We have incorporated the suggestions made by the reviewers. Here is a point-by-point response to the reviewers’ comments and concerns. We note that as part of the changes in response to Reviewers 1 and 3, we have revised the title of the manuscript to “Modelling response strategies for controlling *gonorrhoea* outbreaks in men who have sex with men in Australia”.

# Reviewer #1:

This is a modelling study evaluating the impact of several public health strategies (such as different screening rates combined with subsequent treatment, or different contact tracing levels) on the dissemination of emergent or imported strains of Neisseria gonorrhoeae in Australian MSM. The authors find that the current approach (STI screening 1x/year, unless more than 20 partners/year) will not succeed in eliminating emergent or imported NG strains, unless response strategies are combined (e.g. increased STI screening + contact tracing of casual partners).

The manuscript is well-written, and the methodology seems sound. I have however a few concerns around the applicability, the reporting, and the reproducibility of the study findings.

1. If the aim of this paper is to model different strategies so as to better inform public health authorities and help shaping new guidance, then the model should reflect the current situation in Australia. In the light of the recent, high uptake of PrEP programs (EPIC-NSW or PrEP-X) by a relevant part of the MSM population, I am a bit concerned whether the present model structure really allows up-to-date inferences to be made. The use of PrEP has been suggested to affect several of the model parameters, such as condom use, casual partner acquisition rate or the distribution of the model population into high (27%) and low (73%) risk. It would be thus useful to adapt the model so that it reflects the changes induced by the wide implementation of PrEP programs. As a minimum, I would strongly suggest to update the parameter “casual partner acquisition rate”, which refers to data from the Health in Men study (published more than 10 years ago).

**Response:** We thank the reviewer for the comment here and provide some additional explanation about our approach. We use data from the Gay Community Periodic Survey (GCPS) from 2018 to inform the fraction available for casual partnerships and condom use but it is correct that we use the HIM study to inform casual partner acquisition rates. However, there is a balancing act in our model between partner acquisition rates and probabilities of transmission per act. This is because we calibrate the model to per-site NG prevalence data collected in the ACCESS study (also for 2018). If for instance we increase the casual partner acquisition rates, calibration would simply lead to lower probabilities of transmission per act. This reflects a fundamental limitation in terms of not having sufficient data for all parameters to be identifiable.

We have added the following text in discussion at line 306.

“However, it is possible that rates of transmission may have risen since this time, as pre-exposure prophylaxis for HIV (PrEP) has been funded by the Pharmaceutical Benefits Scheme (PBS) since December 2017 with high uptake among eligible MSM, and evidence of potential changes in sexual risk behaviour associated with this rise according to GCPS Sydney 2018 (23). The impact of PrEP on the spread of NG is unclear, as increases in condom-less intercourse may be balanced by requirements for regular STI testing for receipt of PrEP but future outbreaks may be more difficult to control than our simulations suggest.”

2. Throughout the manuscript (including key words and abstract), there is a lot of emphasis on drug resistant NG strains, so that the reader truly expects a model estimating the effect of different response strategies on a resistant type of NG. Surprisingly, this aim is not further pursued in the analytical part and the reasons given in the discussion seem to reflect some post-hoc decision. It would be very useful to provide a study protocol describing how the analysis was originally planned, and detailing the changes that were made to the initial plan.

**Response**: We recognise that this aspect of the paper has been confusing and have made significant textual changes particularly in the abstract and introduction to clarify that while our interest in these strategies relates to threat of XDR NG, we focus here just on control of a generic imported strain. We note that this was also raised by reviewer 3 as an issue in terms of clarity of aims.

In terms of the general plan for this study and related work – this study was always aimed at this focus on control of a single imported strain. We are currently undertaking data collection to identify likely rates of importation and are in the early stages of looking at a more comprehensive simulation framework in which multiple strains are present, resistance is characterised and we look at implications in terms of resource use etc.

We have made the following text changes:

In line 43, “emergent XDR NG strains” to “imported NG strains”.

In line 49, “emergent XDR NG strains” to “imported NG strains”.

The end of the Introduction (at line 113) is rewritten as follows:

“In this study we develop an anatomic site-specific individual-based model model of gonorrhoea transmission in an urban Australian MSM population. Although similar in some aspects to other site-specific models of NG in MSM (e.g., two Australian studies (18, 20) and two US studies (21, 22)), this model has been specifically developed to examine outbreak control strategies for imported NG infection. Here, we evaluate the potential impact of community and individual-level test-and-treat strategies to control outbreaks of imported NG strains in the Australian MSM population.”

Reference:

[18] Lei Zhang, David G Regan, Eric PF Chow, Manoj Gambhir, Vincent Cornelisse, Andrew Grulich, Jason Ong, David A Lewis, Jane Hocking, Christopher K Fairley. Neisseria gonorrhoeae Transmission Among Men Who Have Sex With Men: An Anatomical Site-Specific Mathematical Model Evaluating the Potential Preventive Impact of Mouthwash, Sexually Transmitted Diseases: 2017, 44(10), p 586-592.

[20] Ben B Hui, David M Whiley, Basil Donovan, Matthew G Law, David G Regan. [Identifying factors that lead to the persistence of imported gonorrhoeae strains: a modelling study](https://sti.bmj.com/content/93/3/221.short), Sexually transmitted infections: 2017, 93(3), p221-225.

[21] Ian H. Spicknall, Kenneth H. Mayer, Sevgi O. Aral, Ethan O. Romero-Severson. Assessing Uncertainty in an Anatomical Site-Specific Gonorrhea Transmission Model of Men Who Have Sex With Men, Sexually Transmitted Diseases: 2019: 46(5), p 321-328.

[22] Earnest R, Rönn MM, Bellerose M, Menon-Johansson AS, Berruti AA, Chesson HW, et al. Modeling the Cost-Effectiveness of Express Multi-Site Gonorrhea Screening among Men Who Have Sex with Men in the United States. Sex Transm Dis. 2021;Publish Ahead of Print.

3. It is not clear if someone can switch from casual partnership to regular partnership, and vice versa once they separate from their last partnership. It is also not clear if the NG strain is imported only once or whether it is a continuous trend? This should be specified. Assuming there is no switching of partnership type, it is a bit surprising to see that partner testing in those with stable partnerships only would also result in a complete extinction of NG strains in those with casual partnerships. One would rather expect an endemic situation in the cluster of those having casual partnerships only. How do you justify this?

**Response:** We thank the reviewer for this comment. As brief responses, no the model does not allowing switching of partnership preferences during simulations. We have added the following text to clarify this at line 383.

“On entry to the population, individuals are assigned a partner-type preference, which does not change over the simulation period.”

In regard to importations, yes this is a single importation event. The question of multiple importations is an important one (also raised by reviewer 3), which we did consider addressing here. However, in practice this requires a more in-depth consideration of various aspects of strain dynamics and interventions that we plan to address in future work.

We have provided a more detailed response to reviewer 3 around this and related questions but note the following changes to the text:

End of limitations paragraph in Discussion at line 313:

“This study also considers just a single importation event, when in practice repeated importation events may occur. Though the model is able to incorporate multiple and concurrent importation events, further development is needed in regard to capture multiple concurrent strains and the capacity of public health responses to address simultaneous outbreaks. In addition, data to inform the rate at which imported NG infections occur is not currently available.”

Finally in regard to persistence of infections in populations with only casual partnerships, within the existing manuscript we note that there is persistence of simulations at 5 years under current testing with testing of regular partners (1.44% at 5 years as shown in Table 3) and a small fraction (0.2%) persist at 5 years even if background testing increased to recommended levels. For more rapid elimination testing of casual partners as well is required as noted in the manuscript.

From a technical point of view, testing of regular partners has a significant impact as only 23% of the MSM population in simulations have only casual partners, and regular partnerships are important in sustaining the imported strain. The imported strain can be introduced to individuals with casual partnerships, but these individuals are very likely to have regular partners and testing regular partners therefore has an impact on transmission. As recommended testing impacts on the whole MSM population it further inhibits transmission in both those with only casual partners.

4. ‘$\text{Pharynx :urethra}\text{: rectum=49:17:34}$’ has been simplified to ‘$\text{pharynx: urethra: rectum}\text{ }\text{=}\text{ }\text{47:23:3}$’. I assume the last number (i.e. 3) is a typo. If not, the simplification would be totally different from the ratio derived from ACCESS database. Please explain.

**Response:** This is a typo in the Technical Appendix and has been corrected to say $\text{pharynx: urethra: rectum=}\text{47:23:30}$.

5. There is no information provided on the program/software used. It is necessary to provide this information for reproducibility. In addition, please provide a link to the source code of your model on a GIT repository.

**Response:** We have now made the source code available via GitHub via the link <https://github.com/qbduan/Gonorrhea-modeling-1>.

And we have also added a sentence on page 2 of the supplementary material as follows:

“The source code for the model implementation in C++ can be found in GitHub (<https://github.com/qbduan/Gonorrhea-modeling-1>).”

6. Figure 1 seems a bit jumbled and hard to understand as there is no transition between the states such as ‘disease transmission’ and ‘recovery’. Also, it is worth mentioning SEIR in bracket in ‘diseases transmission’.

**Response**: Figure 1 illustrates the process of a single model cycle of 1 day and the ordering of events. This is an agent-based model of both partnerships and transmission and so there are a number of event types (queued in sequence) that occur in each cycle. In relation to disease transmission – this reference in the figure only relates to transmission events. Recovery (and loss of short-term immunity) are processed as part of updating the states of all agents in the last stage of the daily simulation cycle.

We have added the following sentence to the caption of Figure 1 to make this clearer.

“In each cycle, the status of each individual and the sexual partnership network are carried over from the previous simulation step. Sexual events are scheduled daily for each partnership, and if disease transmission occurs, infection status is changed for the relevant individual(s). Events relating to natural progression of infection (“Disease transmission” and “Recovery”), testing, treatment of infection (“Screening/Treatment”) and entry/exit of individuals (“Replacement”) from the sexually active population are then processed before concluding each simulation cycle with partnership formation and dissolution for the next simulation cycle.”

7. In table 9, it is unclear why you are presenting the column ‘Calibrated solution (worst)’? Rather provide the best solution and 95% quartile or CI around the best calibrated solution.

**Response**: This reflects our choice of terminology – “worst” here is reflecting worst case assumptions around treatment program parameters. In our study, for the purpose of sensitivity analysis of diagnosis and treatment, we set two different scenarios, i.e., base case scenario where 100% sensitivity, 100% treatment efficacy and clearance of infection from all anatomical sites within 1 day are assumed, and pessimistic scenarios where 95% test sensitivity, 95% treatment efficacy, and 7 days clearance delay for asymptomatic infection and 1 day for symptomatic infection are assumed.

We have revised our wording to remove this confusion and now describe this as the ‘Pessimistic-case’, when we assume that diagnostic sensitivity is 95% and treatment efficacy is 95% for the baseline model. The column names in Table 9 are now ‘base-case’ and ‘pessimistic-case’.

8. Figure A5 of supplementary is exactly the same as Figure 3 panel B and C in the manuscript. I suggest you remove one of them.

**Response**: The two figures do actually represent differing sets of scenarios. In the main text, Figure 3 panels B and C, we plot the following eight scenarios:

1. CT: current testing level.

2. RT: recommended testing level.

3. CT+PTT_R_: contact tracing of 100% regular partners under current testing level.

4. RT+PTT_R_: contact tracing of 100% regular partners under recommended testing level.

5. CT+PTT_RC20_: contact tracing of 100% regular partners + 20% casual partners under current testing level.

6. RT+PTT_RC20_: contact tracing of 100% regular partners + 20% casual partners under recommended testing level.

7. CT+PTT_RC50_: contact tracing of 100% regular partners + 50% casual partners under current testing level.

8. RT+PTT_RC50_: contact tracing of 100% regular partners + 50% casual partners under recommended testing level.

In Technical Appendix Figure A5, we plot eight similar scenarios but with some differences in key assumptions:

1. CT+PTT_R(6-month)_: response strategies of contact tracing of 100% regular partners are delayed for 6 months after the first detection of the imported strain.

2. RT_(6-month)_: response strategies of recommended testing level are delayed for 6 months after the first detection of the imported strain.

3. CT+PTT_R80_: contact tracing of 80% regular partners under current testing level.

4. RT+PTT_R80_: contact tracing of 80% regular partners under recommended testing level.

5. CT+PTT_RC30:_ contact tracing of 100% regular partners + 30% casual partners under current testing level.

6. RT+PTT_RC30_: contact tracing of 100% regular partners + 30% casual partners under recommended testing level.

7. CT+PTT_RC40_: contact tracing of 100% regular partners + 40% casual partners under current testing level.

8. RT+PTT_RC40_: contact tracing of 100% regular partners + 40% casual partners under recommended testing level.

The notation of each strategy has been specified in Section 2.2 of Technical Appendix, and we have added a sentence there to further clarify the meaning of “CT+PTT_R(6-month)_” and “RT_R(6-month)_” as follows.

“We also consider a delay in responses, i.e. a 6-month delay for contact tracing of regular partners under current testing level (CT+PTT_R(6-month)_) and 6-month delay for increasing the testing to recommended level (RT_R(6-month)_).”

# Reviewer #2:

1. The Introduction section must be more detailed. It must contain review of previous works

**Response**: We have added the following text to the end of introduction at line 113.

“In this study we develop an anatomic site-specific individual-based model model of gonorrhoea transmission in an urban Australian MSM population. Although similar in some aspects to other site-specific models of NG in MSM (e.g., two Australian studies (18, 20) and two US studies (21, 22)), this model has been specifically developed to examine outbreak control strategies for imported NG infection. Here, we evaluate the potential impact of community and individual-level test-and-treat strategies to control outbreaks of imported NG strains in the Australian MSM population.”

Reference:

[18] Lei Zhang, David G Regan, Eric PF Chow, Manoj Gambhir, Vincent Cornelisse, Andrew Grulich, Jason Ong, David A Lewis, Jane Hocking, Christopher K Fairley. Neisseria gonorrhoeae Transmission Among Men Who Have Sex With Men: An Anatomical Site-Specific Mathematical Model Evaluating the Potential Preventive Impact of Mouthwash, Sexually Transmitted Diseases: 2017, 44(10), p 586-592.

[20] Ben B Hui, David M Whiley, Basil Donovan, Matthew G Law, David G Regan. [Identifying factors that lead to the persistence of imported gonorrhoeae strains: a modelling study](https://sti.bmj.com/content/93/3/221.short), Sexually transmitted infections: 2017, 93(3), p221-225.

[21] Ian H. Spicknall, Kenneth H. Mayer, Sevgi O. Aral, Ethan O. Romero-Severson. Assessing Uncertainty in an Anatomical Site-Specific Gonorrhea Transmission Model of Men Who Have Sex With Men, Sexually Transmitted Diseases: 2019: 46(5), p 321-328.

[22] Earnest R, Rönn MM, Bellerose M, Menon-Johansson AS, Berruti AA, Chesson HW, et al. Modeling the Cost-Effectiveness of Express Multi-Site Gonorrhea Screening among Men Who Have Sex with Men in the United States. Sex Transm Dis. 2021;Publish Ahead of Print.

2. The authors must clearly state whether the model is new or whether they're modifying an existing model

**Response**: We have added some text to the end of introduction at line 113 as for comment point 1.

“In this study we develop an anatomic site-specific individual-based model model of gonorrhoea transmission in an urban Australian MSM population. Although similar in some aspects to other site-specific models of NG in MSM (e.g., two Australian studies (18, 20) and two US studies (21, 22)), this model has been specifically developed to examine outbreak control strategies for imported NG infection. Here, we evaluate the potential impact of community and individual-level test-and-treat strategies to control outbreaks of imported NG strains in the Australian MSM population.”

3. The model equations should be presented.

**Response**: As is described clearly in the paper and Technical Appendix, our model is a stochastic individual-based simulation model. As partnership formation follows an algorithmic process, it is not possible to define an equation-based description of model behaviour and instead we do this via detailed descriptions of the algorithmic processes in the Technical Appendix. We have also made model code available on GitHub in response to comments from reviewer 1 above. The model is represented by a dynamic network of sexually active individuals that are linked by sexual partnerships, rather than equations. The relevant parameters regarding the formation and separation of sexual partnerships, schedule of sexual activities, disease progression and transmission are either informed by published literature or derived from model calibration. The natural history of gonorrhoea is captured in a Susceptible-Exposed-Infectious-Recovered (SEIRS) framework.

4. The results and discussion must clearly show how this model improved on previous ones.

**Response**: We have added the following text to the end of discussion at line 335.

“In terms of technical advances over previous studies, our model reflects recent evidence of the importance of kissing as a transmission route (pharynx to pharynx) of NG in capturing the high observed prevalence and incidence of oropharyngeal infections. Secondly, we include group sex events as a subset of casual partnerships, reflecting real-world behavioural data. Finally, by explicitly linking to importations and integrating surveillance components, the model provides a framework for a more complete assessment of importation risk and strategies to control resistant NG infections.”

# Reviewer #3:

In their manuscript, Duan et al use an individual-based, anatomical site-specific mathematical model of Ng transmission to ask the question: what types of testing and treatment strategies would be needed if an XDR Ng strain were introduced in the Australian MSM population? They highlighted several strategies that would be able to mitigate spread/eradicate circulation of this strain, namely combining case-based and population-based strategies (nicely summarized in Table 3). The model is well constructed and will certainly be useful for others examining specific questions on Ng transmission. There are, however, some concerns that need to be addressed.

1. The most obvious concern is how this model relates to the spread of XDR Ng. The authors assume that if such a strain is introduced, it can be treated just as well as any other Ng strain (ln 148-149). The real supposed concern with XDR Ng is that no treatment options will be available for these infections, meaning increased risk of spread. I do agree with the authors that other treatment alternatives could be used to cure Ng, but there will be delay in curing Ng in these individuals. The authors need to incorporate some aspects of treatment efficacy and duration of unsuccessful treatment if they want to infer anything on resistant variants circulating in the population. Furthermore, XDR Ng could naturally develop within the MSM population in Australia. Although rare, it could happen and might also affect the scenarios modeled. If the authors cannot address specific questions of resistance, they need to place their focus on Ng transmission rather than Ng resistance.

**Response**: We recognise the confusion around the focus of the paper, which was also identified by reviewer 1. Here we have revised our description to make it clear that the focus is on controlling imported NG infection, rather than specifically XDR infection in this paper. As noted above in comments to reviewer 1, there is some additional data collection and model development required to adequately deal with resistant strains co-circulating with wild-type and the aim is to address these questions in future work. We replicate the relevant changes described above in responses to reviewer 1 here that we believe help clarify the focus of this paper to the reader. In regard to the comment about de novo resistance – we agree that this could occur (although is unlikely for ceftriaxone) and intend to consider this in future work (this should not require significant changes to the intervention model).

We have made the following text changes:

In line 43, “emergent XDR NG strains” to “imported NG strains”.

In line 49, “emergent XDR NG strains” to “imported NG strains”.

The end of Introduction is rewritten as follows:

“In this study we develop an anatomic site-specific individual-based model of gonorrhoea transmission in an urban Australian MSM population. Although similar in some aspects to other site-specific models of NG in MSM (e.g., two Australian studies (18, 20) and two US studies (21, 22)), this model has been specifically developed to examine outbreak control strategies for imported NG infection. Here, we evaluate the potential impact of community and individual-level test-and-treat strategies to control outbreaks of imported NG strains in the Australian MSM population.”

Reference:

[18] Lei Zhang, David G Regan, Eric PF Chow, Manoj Gambhir, Vincent Cornelisse, Andrew Grulich, Jason Ong, David A Lewis, Jane Hocking, Christopher K Fairley. Neisseria gonorrhoeae Transmission Among Men Who Have Sex With Men: An Anatomical Site-Specific Mathematical Model Evaluating the Potential Preventive Impact of Mouthwash, Sexually Transmitted Diseases: 2017, 44(10), p 586-592.

[20] Ben B Hui, David M Whiley, Basil Donovan, Matthew G Law, David G Regan. [Identifying factors that lead to the persistence of imported gonorrhoeae strains: a modelling study](https://sti.bmj.com/content/93/3/221.short), Sexually transmitted infections: 2017, 93(3), p221-225.

[21] Ian H. Spicknall, Kenneth H. Mayer, Sevgi O. Aral, Ethan O. Romero-Severson. Assessing Uncertainty in an Anatomical Site-Specific Gonorrhea Transmission Model of Men Who Have Sex With Men, Sexually Transmitted Diseases: 2019: 46(5), p 321-328.

[22] Earnest R, Rönn MM, Bellerose M, Menon-Johansson AS, Berruti AA, Chesson HW, et al. Modeling the Cost-Effectiveness of Express Multi-Site Gonorrhea Screening among Men Who Have Sex with Men in the United States. Sex Transm Dis. 2021;Publish Ahead of Print.

2. Another major assumption, from what I understand, is that only one introduction is expected and the resulting transmission patterns stem from this single introduction (ln 144-146). Given the worldwide network of MSM contacts and STI transmission, there would likely be several introductions of the XDR Ng strain in the population, which could have an impact on eradication of XDR Ng. These aspects also need to be considered in the model.

**Response**: As pointed out by the reviewer, this is a major assumption. Clearly in practice, we will be exposed to repeated importation events. We did seriously consider including additional results in relation to the question but in the end abandoned these efforts as it would have required reshaping the entire paper and sits much more as an aim of future work we are planning.

In terms of reasons for not pursuing this further here – in particular.

(1) We are currently analysing data that will help us inform realistic estimates of importation rates for NG strains to Australia.
(2) We believe that some measure of public effort associated with investigations is required, as these responses are subject to resource constraints and performance likely declines with multiple concurrent outbreaks.
(3) We are investigating the utility of genomic surveillance in regard to this question.
(4) Integrating these various aspects will take considerable time (i.e. 6-12 months) and we feel that there is sufficient value in this study as a single importation test case for it to stand alone.

However, we do want to stress that this is clearly an important caveat of the work and a critical step in terms of translation to a public health response framework.

We have added the following text to the end of limitation paragraph in Discussion at line 313:

“This study also considers just a single importation event, when in practice repeated importation events may occur. Though the model is able to incorporate multiple and concurrent importation events, further development is needed in regard to capture multiple concurrent strains and the capacity of public health responses to address simultaneous outbreaks. In addition, data to inform the rate at which imported NG infections occur is not currently available.”

3. It is also unclear whether the model incorporates the fact that the prevalence of Ng infections in MSM has been steadily increasing. Similarly, condomless anal sex has also increased in several studies among MSM. The inputs used in this model are calibrated to 2018 levels, but does an epidemic background of increasing Ng prevalence and more frequent condomless anal sex render these strategies less effective?

**Response**: A similar question was raised by reviewer 1, to which we have responded to above. We note in addition that the model is calibrated to the NG prevalence in 2018 which was the most recent data we have access to. It is unclear in the Australian context whether there has been a continued rise (and certainly the 2018 data captures a significant period of PrEP uptake and potential behaviour change). NG cases nationally have recently declined due to COVID restrictions (both on travel and via local restrictions). However, we recognise that calibration to a flat prevalence level as opposed to an increasing trend might underestimate the underlying transmission parameters. We have added the following sentence in discussion section (line 296).

“However, it is possible that rates of transmission may have risen since this time, as pre-exposure prophylaxis for HIV (PrEP) has been funded by the Pharmaceutical Benefits Scheme (PBS) since December 2017 with high uptake among eligible MSM, and evidence of potential changes in sexual risk behaviour associated with this rise according to GCPS Sydney 2018 (23). The impact of PrEP on the spread of NG is unclear, as increases in condom-less intercourse may be balanced by requirements for regular STI testing for receipt of PrEP but future outbreaks may be more difficult to control than our simulations suggest.”

To clarify the impact of condom use in our study, we have also added the following two texts at line 235 and line 293.

“The outcomes from simulations were insensitive to changes in condom-use in the base-case scenario due to the dominant role of oro-pharyngeal transmission in our model. We also examined a scenario with a reduced symptomatic urethral proportion of 60% that, after recalibration, leads to a greater role of urethral transmission in sustaining prevalence. In this scenario increases in condom-use become an effective additional means of controlling NG outbreaks under CT (Table A13).”

“However, if we assume a lower but potentially unrealistic symptomatic urethral infection proportion of 60% we found that increasing condom-use has additional benefit in controlling NG outbreaks.”

Minor comments:
- ln 46. Should this be “current _testing and treatment_ practices”?

**Response**: Here ‘current practice’ means the current level of interventions, i.e., current testing levels and no contact tracing for NG infections. We have changed the wording as “current practice (current testing levels and no contact tracing)” in manuscript.

- ln 115. Are the authors really testing “surveillance” strategies? Is Ng required to be reported to the Australian government?

**Response**:

- Yes, we think this is fair description, although the boundary between enhanced surveillance and outbreak response is not sharp. We have rewritten the text at the end of the Introduction at line 117 as follows:

“Here, we evaluate the potential impact of community and individual-level test-and-treat strategies to control outbreaks of imported NG strains in the Australian MSM population.”

- Yes, NG is a notifiable disease in Australia and NG notification data is collected by states and territories and reporting on by the Australian government as well in annual STI reporting by the Kirby Institute. AMR is monitored by Australian Gonococcal Surveillance program (between 25 and 30% of NG positive tests have a sample taken for culture and AMR testing).

- ln 116-7. Not sure how the sentence “note that this…” relates to this paragraph.

**Response**: We have deleted this sentence here.

- ln 168-170. How close are these inputs to the current situation?

**Response**: We do not have data to inform the current level of contact tracing in Australia MSM in relation to outbreaks. Current testing (CT) reflects the recent data on testing frequency among Australian MSM. We have modified the text as follows at line 157.

“…, 1) current testing (CT) reflects testing coverage as reported in GCPS Sydney 2018 (23) and The Australian Collaboration for Coordinated Enhanced Sentinel Surveillance of Blood Borne Viruses and Sexually Transmitted Infections (ACCESS) (26); …”

- ln 177. The 2.3% prevalence refers to any site? Can this be divvied out by site?

**Response**: Yes, this prevalence refers to any site. This can be divided out by site, but we have not presented intermediate site-specific prevalence. When it comes to 5 years post-importation under current testing level, the infection prevalence is ~14.7%, while the site-specific prevalence would fluctuate around the prevalence target, i.e., 8.6% at oropharynx, 8.3% at anorectum and 0.26% at urethra. We have modified the text in line 185 as follows.

“…, with mean population prevalence of 2.3% (IQR [0.5%-3.6%]) at any anatomical site.”

- ln 361. By how much is the estimate missed when not included in this study?

**Response**: According to our calibration results, nearly 90% of oropharyngeal infections originate from kissing. This number is even higher in some other modelling studies in MSM, e.g. see Zhang et al 2017. If kissing were not included, a large part (~70%) of these infections would not occur even if the per act transmission probabilities of urethra and rectum to oropharynx are increased to 1. In the case of no kissing, other parameters must be greatly increased in order to match the observed level of oropharyngeal infections. For example, either the prevalence of urethral infection needs to increase ~5-fold above the calibration target (to ~1.3%), the asymptomatic proportion for urethral infection needs to increase from 10% to 70%, or the frequency of oral sex is significantly increased by ~5-fold. With such a change in parameters, we can match the target level of oropharyngeal infections, but these parameters/output values are inconsistent with reality. We have added the following text in Technical Appendix above Table A5.

“Kissing is an important transmission routine for oropharyngeal infection [20, 21], and if it is not included, the model is not able to reproduce the high prevalence of oropharyngeal infection under the assumed level of probability and frequency of kissing in Table A5. As noted above, in the case of no kissing, other parameters must be greatly increased in order to match the observed level of oropharyngeal infections. For example, either the prevalence of urethral infection needs to increase ~5-fold above the calibration target (to ~1.3%), the asymptomatic proportion for urethral infection needs to increase from 10% to ~70%, or the frequency of oral sex is significantly increased by ~5-fold. With such a change in parameters, we can match the target level of oropharyngeal infections, but these parameters/output values are inconsistent with reality.”

Reference

Lei Zhang, David G Regan, Eric PF Chow, Manoj Gambhir, Vincent Cornelisse, Andrew Grulich, Jason Ong, David A Lewis, Jane Hocking, Christopher K Fairley. *Neisseria gonorrhoeae* Transmission Among Men Who Have Sex With Men: An Anatomical Site-Specific Mathematical Model Evaluating the Potential Preventive Impact of Mouthwash, Sexually Transmitted Diseases: October 2017 - Volume 44 - Issue 10 - p 586-592.

- Figure 1. It would be helpful to have blocks regrouping events that are computed within the same algorithm.

**Response**: We have added the following sentence to the caption of Figure 1 to make this clearer.

“In each cycle, the status of each individual and the sexual partnership network are carried over from the previous simulation step. Sexual events are scheduled daily for each partnership, and if disease transmission occurs, infection status is changed for the relevant individual(s). Events relating to natural progression of infection (“Disease transmission” and “Recovery”), testing, treatment of infection (“Screening/Treatment”) and entry/exit of individuals (“Replacement”) from the sexually active population are then processed before concluding each simulation cycle with partnership formation and dissolution for the next simulation cycle”

- Table 1. Missing “(“ in the second column, row “Anorectum”.

**Response**: We have added “(” for that row.

- Technical appendix: Algorithm 1, step 3. Why does the % infected at the sites not match those listed on lines 132-133?

**Response:** The values in Algorithms 1 are the initial proportions of site-specific infections to start the transmission simulation, when the multiple-site co-infections and partnership status are not considered. We intended to run the model for a burn-in period of time, so that distribution of site-specific infection and multiple-site co-infections can reach the equilibrium state. And after the burn-in period, the site-specific prevalence reverts to the targeting prevalence listed on lines 132-133. The initial values would not affect the equilibrium state of the system.

We have added a footnote to Algorithm 1 in Technical Appendix as follows:

“Note: 7%, 7% and 0.5% in step 3 are used as initial values for proportions of site-specific infections to start the transmission simulation of local strains. Use of other reasonable values for these initial conditions does not affect the equilibrium prevalences that simulations fluctuate around.”

- Technical appendix: There are two “Algorithm 3” in the manuscript.

**Response:** We have renumbered all algorithms and corrected the corresponding reference.

- I would highly recommend the authors place the data and programming code on a publicly available server.

**Response:** As noted above in our response to reviewer 1, we have now We provided the source code in GitHub via the link <https://github.com/qbduan/Gonorrhea-modeling-1>. We have also added a sentence in supplementary page 2 as follows:

“The source code of the model implementation in C++ can be found in GitHub (<https://github.com/qbduan/Gonorrhea-modeling-1>).”
